# Circulating intermediate monocytes CD14++CD16+ are increased after elective percutaneous coronary intervention

**DOI:** 10.1371/journal.pone.0294746

**Published:** 2023-12-14

**Authors:** Ioannis Merinopoulos, U Bhalraam, Terri Holmes, Vasiliki Tsampasian, Natasha Corballis, Tharusha Gunawardena, Chris Sawh, Clint Maart, Trevor Wistow, Alisdair Ryding, Simon C. Eccleshall, James Smith, Vassilios S. Vassiliou

**Affiliations:** 1 Department of Cardiology, Norfolk and Norwich University Hospital, Norwich, United Kingdom; 2 Norwich Medical School, University of East Anglia, Norwich, United Kingdom; 3 Institute of Continuing Education, University of Cambridge, Cambridge, United Kingdom; University of Texas Medical Branch at Galveston, UNITED STATES

## Abstract

**Aim:**

Inflammation plays a central role in the pathogenesis of atherosclerosis and in the sequelae of percutaneous coronary intervention (PCI). Previous work demonstrated that intermediate monocytes (CD14++CD16+) are associated with adverse cardiovascular events, yet monocyte subset response following elective PCI has not been described. This article explores the changes in monocyte subset and humoral response after elective PCI.

**Methods:**

This prospective study included 30 patients without inflammatory diseases being referred for elective PCI. We included patients treated with drug coated balloons or 2^nd^ generation drug eluting stents. Patients underwent blood tests at baseline (prior to PCI), four hours, two weeks and two months later. Analyses were performed in terms of monocyte subsets (classical CD14++CD16-, intermediate CD14++CD16+ and non-classical CD14+CD16++), gene expression of CD14+ leucocytes and humoral biomarkers.

**Results:**

Intermediate monocytes decreased significantly four hours after PCI, were recovered at two weeks, and increased significantly at two months post elective, uncomplicated PCI. They remain significantly elevated in the DES group but not in the DCB group. Gene expression analysis of CD14+ leucocytes showed IL18 had decreased expression at two weeks, CXCR4 and IL1β decreased at two months, while pentraxin 3 increased at two weeks and two months. In terms of humoral biomarkers, hsTnI remains elevated up to two weeks post PCI while IL6 and TNFα remain elevated till two months post PCI.

**Conclusion:**

Intermediate monocytes increase significantly two months following elective, uncomplicated PCI. They remain significantly elevated in the DES group but not in the DCB group suggesting that the PCI strategy could be one of the ways to modulate the inflammatory response post PCI.

## Introduction

It is well established that inflammation plays a central role in the pathogenesis of atherosclerosis but also in the sequelae of percutaneous coronary intervention (PCI) [1,2]. It is increasingly recognised that periprocedural inflammation is associated with worse adverse cardiovascular events [3]. Various inflammatory biomarkers and mediators elicited following PCI, such as C-reactive protein (CRP), pentraxin-3 (PTX3), interleukins (IL), tumour necrosis factor α (TNFα) and leucocytes, have been shown to be associated with worse patient outcomes [4]. Anti-inflammatory and immunomodulatory medications are now being trialled to improve prognosis with encouraging results [5,6]. Monocytes play a crucial role in all stages of atherogenesis, from the initial formation of atherosclerotic plaques to the acute inflammatory phase following plaque destabilisation and finally during myocardial healing and remodelling following myocardial infarction [7]. The relationship of circulating monocytes with in-stent neointimal hyperplasia after bare metal stent implantation was first demonstrated almost 20 years ago [8].

Over the last decade, the nomenclature of distinct monocyte subtypes has been standardised into classical CD14++CD16- monocytes, intermediate CD14++CD16+ monocytes and non-classical CD14+CD16++ monocytes [9]. The classical monocytes are the most abundant subset both in blood (about 80–85% of circulating blood monocytes) but also in atherosclerotic plaques. They express CCR2, CD62L and CD64 and are considered inflammatory mediators [7]. The non-classical monocytes express high levels of CX3CR1 but not CCR2 or CD62L, have patrolling properties and also have important role in angiogenesis [7]. The most recently described intermediate monocytes can be differentiated from non-classical monocytes as they express CCR2. They are the main producer of reactive oxygen species while the receptors they express indicate their pro-atherosclerotic capabilities [7,10]. As the classification of the subsets of monocytes was standardised only in 2010, it is difficult to draw definitive conclusions about the role of various monocytes subsets from older studies.

Linked to inflammation, stent characteristics represent another important mechanism involved in adverse reaction to stents [11]. Foreign body reactions to the metal platform as well as hypersensitivity reactions to the polymer contribute to the inflammation elicited after PCI and have been associated with adverse cardiac events [11]. Previous work has demonstrated that the inflammatory reaction in terms of platelet and neutrophil activation is less after balloon angioplasty compared to bare metal stent implantation [12]. Drug coated balloon (DCB) is an emergent technology allowing drug delivery to the vessel wall without implantation of a permanent scaffold [13]. There is currently no data comparing 2^nd^ generation drug eluting stents (DES) with DCB in terms of the elicited inflammation.

In the present study, we aimed to investigate the cellular as well as the humoral inflammatory response following PCI in the modern era. We assessed the effect of PCI in the acute and short-term on serially measured monocyte subsets and humoral mediators of inflammation. We included patients treated with 2^nd^ generation DES or DCB aiming to compare these PCI strategies in terms of their elicited inflammatory response.

## Methods

### Study population

In this prospective study we recruited adult patients with stable angina undergoing elective PCI for de novo coronary artery disease, either with DES or DCB utilised at the discretion of the operator. We excluded patients with significant renal impairment (estimated glomerular filtration rate <30mL/min/1.73m^2^) or any significant inflammatory condition on immunosuppression as well as pregnant women. Patients treated with DCB should not have more than type B dissection or >30% recoil as per study protocol (in accordance with international consensus [14]). All patients provided written, informed consent prior to being recruited in the study. The study is compliant with the Declaration of Helsinki with regards to an investigation in humans and it was approved by the East of England–Cambridge Central Research Ethics Committee (REC: 19/EE/0075). Patients were identified from outpatient clinics and the elective PCI waiting list of Norfolk & Norwich University Hospital.

### Blood sampling and processing

Patients underwent blood tests at baseline (pre-PCI), four hours, two weeks and two months later. The baseline blood tests were taken from the radial artery sheath prior to intervention and subsequent blood tests were taken by venepuncture. Blood collected from patients in a 5ml serum separator tube (SST) to yield serum aliquots, a 6ml lithium heparin tube to yield plasma aliquots and two 4ml ethylenediaminetetraacetic acid (EDTA) for cellular analysis. The samples were processed once the blood had clotted in the SST tube, within two hours of blood collection and subsequently stored at -80°C until biomarker analysis at the end of the study.

### Cytometric analysis

Human peripheral blood mononuclear cells were isolated and fixed initially until cell staining and flow cytometry within two weeks from blood collection. The antibodies used in this study were FITC anti-human CD14 and APC anti-human CD16 both from Biolegend. Flow cytometry was performed using the CytoFLEX Flow Cytometer (Beckman Coulter, Brea California, United States) and analysis was carried out using FlowJo version 10 software.

### Reverse transcriptase quantitative polymerase chain reaction

CD14 magnetic beads were used to isolate CD14+ leucocytes. Ribonucleic acid (RNA) was isolated from CD14+ leucocytes and quantified. Complimentary deoxyribonucleic acid (cDNA) was synthesized, and quantitative polymerase chain reaction (qPCR) was performed for IL-10, CCL2, CXCR4, TNFα, TREM1, PTX3, CD36, IL-18 and IL-1B at baseline, two weeks and two months post PCI. Cp values were estimated for each sample (values >35 were disregarded as non-specific) and converted to values expressing the fold change from baseline according to the delta-delta method, standardised for a housekeeper gene. Each reaction was repeated three times and an average of the three values was calculated. Real time quantitative PCR was performed on a Roche Lightcycler 480 (Roche, Basel, Switzerland) using SYBR-green technology (PCR biosystems, UK). Supplementary material shows the sequences of the primers used.

### Biomarker analysis

Biomarker analysis for high-sensitivity CRP (hs CRP), high-sensitivity troponin I (hs Trop I), pentraxin-3, IL-6, IL-1β, IL-10 and TNFα was undertaken at the end of the study. Hs CRP and hs Trop I were measured on the Siemens Dimension EXL autoanalyzer. Pentraxin-3, IL-6, IL-1 β, IL-10 and TNFα were measured using assay kits from mesoscale Discovery. These immunoassay kits are run in the enzyme linked immunosorbent assay (ELISA) format but use electrochemiluminescence detection rather than the production of a coloured product.

### Statistical analysis

A visual density-curve inspection and Shapiro-Wilk tests were used to determine normality. If normally distributed, continuous variables were expressed as mean ± standard deviation. Continuous variables that were not normally distributed were expressed as median (interquartile range). Wilcoxon rank-sum and Mann-Whitney U test were used to compare differences in variables between DCB and DES. Differences across the timeframes were assessed pairwise with the Friedman test. The nested p-values were calculated with the Durbin-Conover test after applying Homes adjustments. Categorical variables were compared using the chi-square test. As pairwise analysis can only be performed with complete data, participants with missing data were excluded listwise. All statistical analysis was performed using R Statistical Software (version 2.14.0; R Foundation for Statistical Computing, Vienna, Austria).

## Results

**Fig 1:**
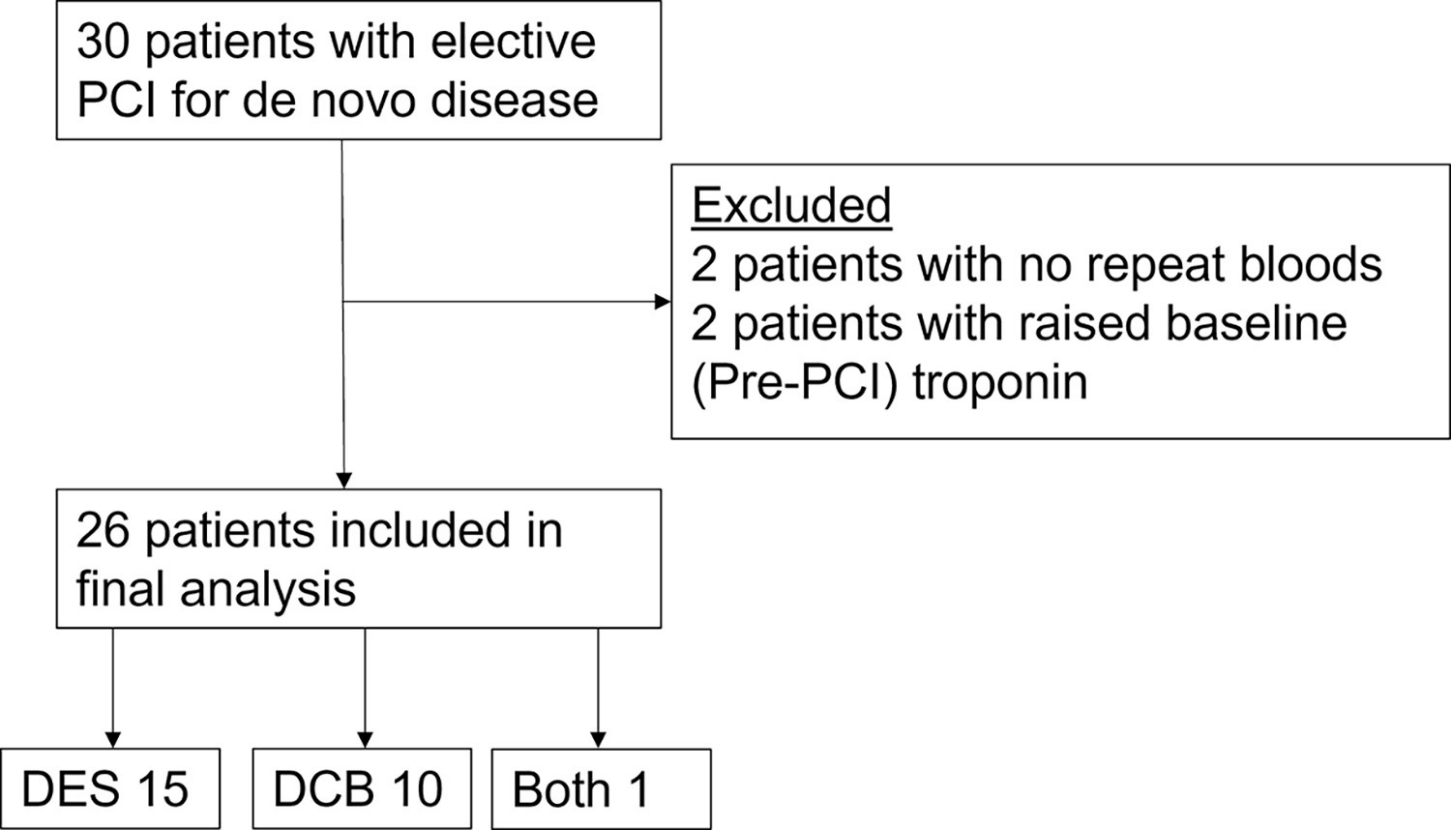
Consort diagram showing the flow of patients in the study.

We recruited 30 patients from June 2020 until July 2021. Two patients did not return for repeat blood tests, two patients had elevated baseline (pre-PCI) troponin (Fig 1) and were thus excluded as per our *a priori* protocol. Following exclusion of these patients 26 patients were included in the final analysis (10 patients treated with DCB, 15 patients treated with DES and one patient treated with both). Their baseline characteristics are shown in Table 1, while the baseline medications are shown in supplementary material and the procedural characteristics in supplementary material.

**Table 1:** Baseline patient characteristics.

	All patients (N = 26)	DCB group N = 10)	DES group (N = 15)	P value
**Age (years)**	69.5 (10.3)	71.6 (11.1)	68.4 (10.1)	0.46
**Male**	23 (88.5%)	9 (90%)	13 (87.7%)	0.8
**Body mass index**	27 (3.8)	26.8 (3.8)	26.7 (3.7)	0.92
**Previous MI**	11 (42.3%)	3 (30%)	8 (53.3%)	0.25
**Previous PCI**	13 (50%)	5 (50%)	8 (53.3%)	0.87
**Diabetes**	5 (19.2%)	2 (20%)	3 (20%)	1
**Stroke**	0	0	0	n/a
**Hypertension**	12 (46.2%)	6 (60%)	6 (40%)	0.32
**Peripheral vascular disease**	0	0	0	n/a
**Atrial fibrillation**	3 (11.5%)	1 (10%)	2 (13.3%)	0.8
**Hypercholesterolaemia**	20 (76.9%)	7 (70%)	12 (80%)	0.56
**Chronic kidney disease (eGFR<60 ml/min/m** ^**2**^ **)**	5 (19.2%)	3 (30%)	2 (13.3%)	0.31
**Mean eGFR ml/min/m** ^**2**^	78.5 (21.1)	70.5 (18.1)	84.1 (22.5)	0.12
**Rheumatoid arthritis**	0	0	0	n/a
**Asthma**	2 (7.7%)	1 (10%)	1 (6.7%)	0.76
**Chronic obstructive pulmonary disease**	2 (7.7%)	1 (10%)	1 (6.7%)	0.76
**Family history of IHD**	9 (35%)	1 (10%)	8 (53.3%)	**0.03**
**Ever smoker**	11 (42.3%)	5 (50%)	6 (40%)	0.62

### Cytometric analysis of monocyte subsets

**Fig 2:**
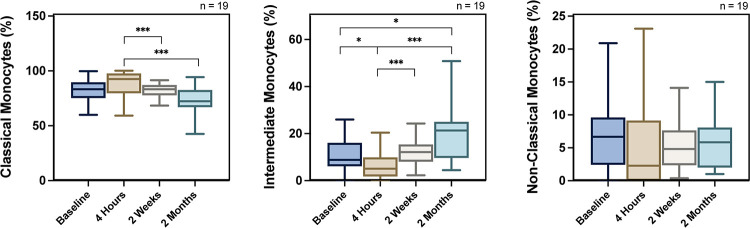
Monocyte response after elective percutaneous coronary intervention, demonstrating the monocyte response for the classical, intermediate and non-classical monocyte subsets. * p<0.05 ** p<0.01 *** p<0.001.

Analysis of the full cohort showed that the intermediate monocytes decreased significantly from baseline to four hours, recovered at two weeks and increased significantly at two months post PCI. In detail, the intermediate monocytes (CD14++CD16+) were 9.07% (7.27–15.9) at baseline, 4.62% (2.39–9.75) at four hours, 12.4% (9.47–16) at two weeks and 21.3% (9.65–25) at two months (Fig 2). Consequently, the opposing trend was seen in classical monocytes (CD14++CD16-). In detail, the percentage of the classical monocytes were 82.4% (75.5–88.7) at baseline, 91.9% (80.3–96.7) at four hours, 82.9% (74.3–86.2) at two weeks and 72.3% (66.9–82.5) at two months (Fig 2). The percentage of classical monocytes at two weeks and two months were significantly reduced compared to four hours but not the baseline. The non-classical monocytes (CD14+CD16++) did not change significantly at any point in time (Fig 2). Analysis of the DCB and DES groups separately demonstrated that there are few differences in the pattern of monocyte response post-PCI between DCB and DES. In the DES group the intermediate monocytes decreased significantly at four hours, recovered at two weeks and increased significantly at two months when compared to baseline (S1 File). In the DCB group there was no difference in the intermediate monocytes at any point when compared to baseline, but they were significantly increased at two weeks and two months when compared to four hours (S1 File). Comparison of the percent change of intermediate monocytes at 2 months from baseline ((intermediate monocytes at 2 months minus intermediate monocytes at baseline) / (intermediate monocytes at baseline)) between DCB and DES revealed a trend towards a significant difference (0.47 ± 0.97 vs 1.81 ± 0.74, p = 0.06). In the DES group the classical monocytes increased significantly at four hours while in the DCB group there was no significant change from baseline at any point (S1 File). Raw data are presented in supplementary material.

### Gene expression of CD14+ monocytes

**Fig 3:**
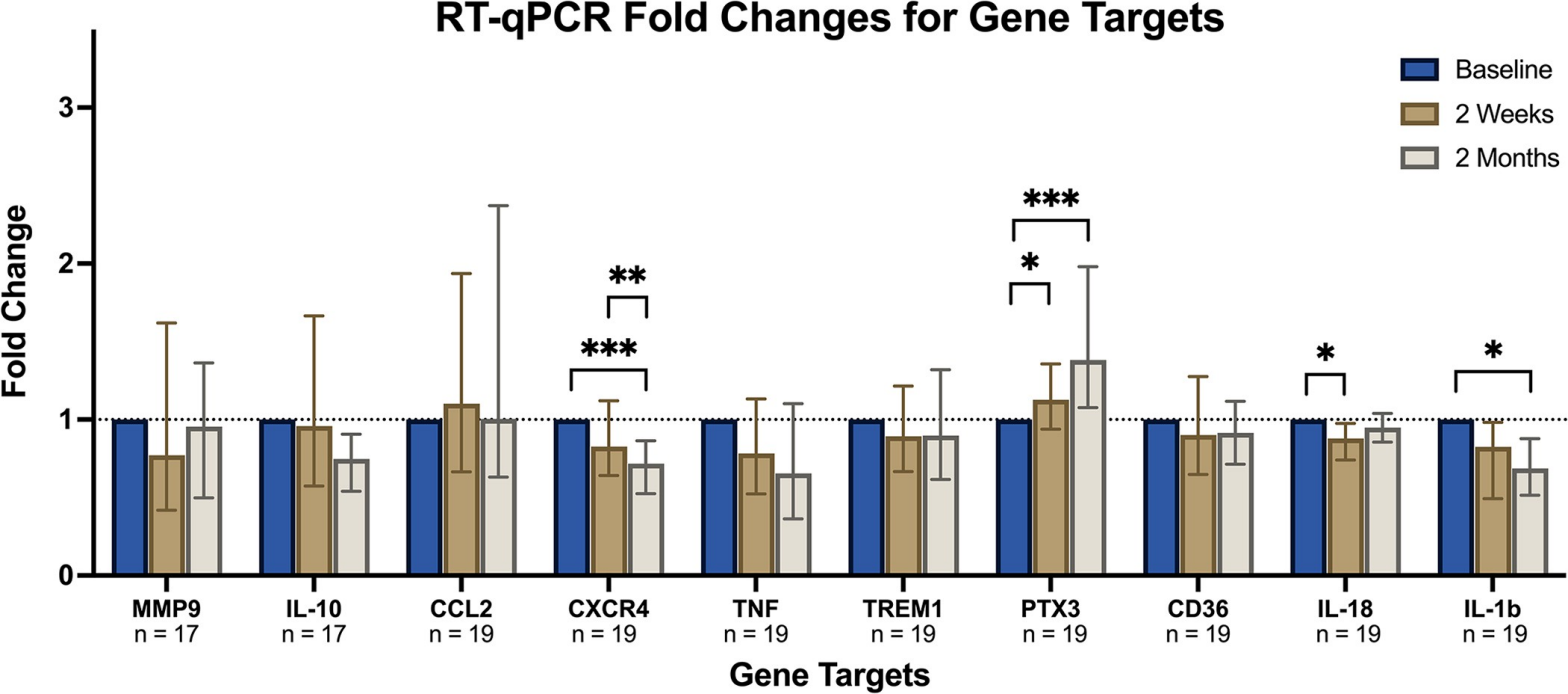
Gene expression of CD14+ leucocytes following elective percutaneous coronary intervention, showing the change in gene expression (fold change compared to baseline) of CD14+ leucocytes following elective angioplasty. * p<0.05 ** p<0.01 *** p<0.001.

Analysis of the full cohort showed that CD14+ leucocytes had a) significantly decreased expression of CXCR4 at two months b) significant increased expression of pentraxin 3 at two weeks and two months c) significantly decreased expression of IL-18 at two weeks and d) significantly decreased expression of IL-1B at two months (Fig 3). Analysis of the DCB and DES groups separately, demonstrated some differences between the groups. In the DCB group, there was a significant decrease of IL-10 expression at two months while there was no significant difference in the DES group. In the DES group, there was a significant decrease of the expression of IL18 and IL-1B at two weeks and two months, while there was no difference in the DCB group (S1 File). Raw data are presented in supplementary material.

### Biomarker analysis

**Fig 4:**
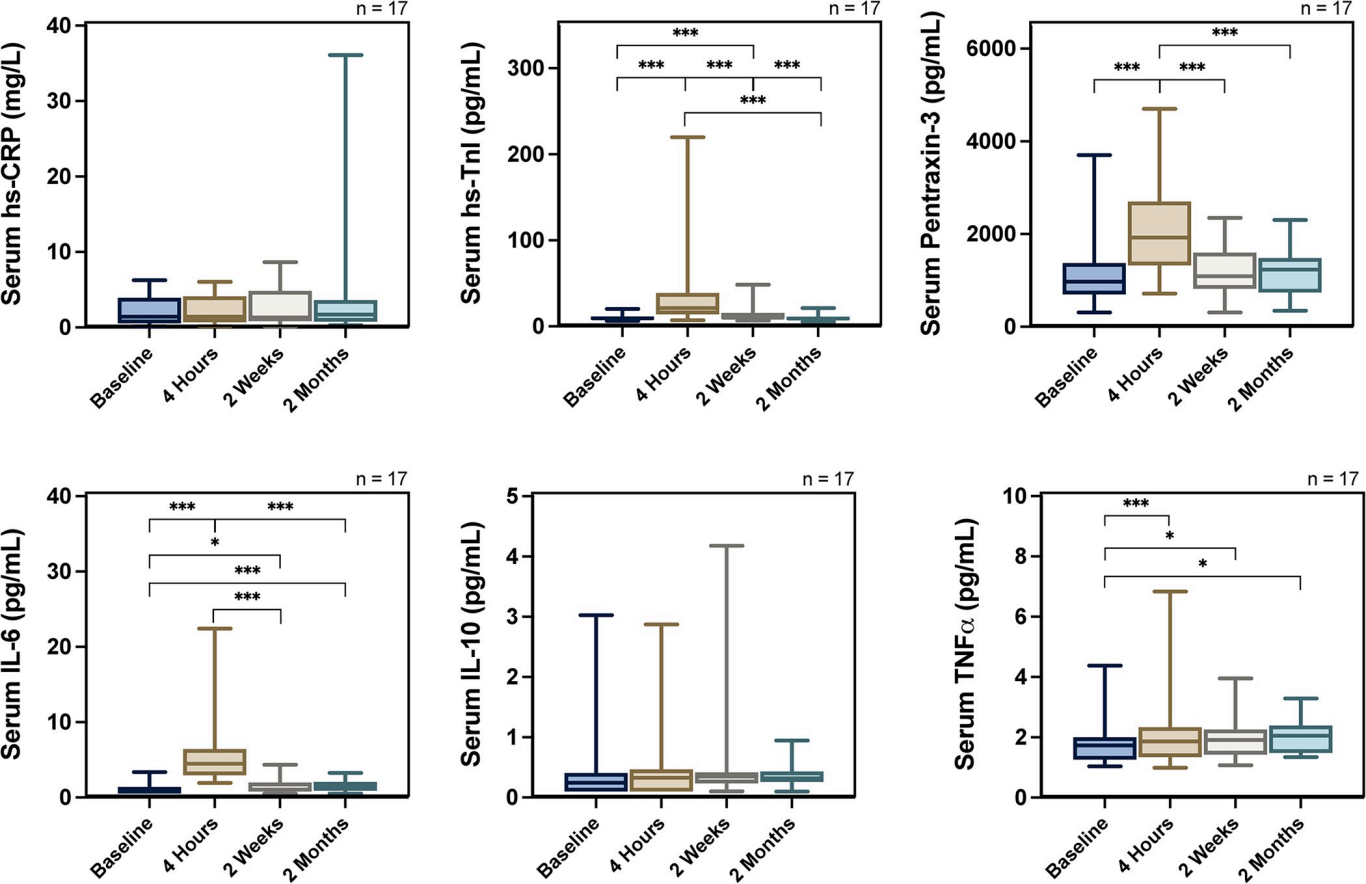
Inflammatory biomarker response following elective percutaneous coronary intervention, showing the inflammatory biomarker response following elective percutaneous coronary intervention. * p<0.05 ** p<0.01 *** p<0.001.

Analysis of the full cohort showed that a) both IL-6 and TNF-α peaked at four hours and remained significantly elevated post-PCI until two months later, b) hsTroponin I peaked at four hours and remained significantly elevated until two weeks later c) Pentraxin 3 was significantly elevated only at four hours and d) there was no significant difference at hsCRP or IL-10 at any point in time (Fig 4). Analysis of the DCB and DES group separately demonstrated only few differences between the groups. In the DCB group, hsTroponin I was significantly elevated only at four hours while in the DES group it remained significantly elevated until two weeks later. In the DCB group, pentraxin 3 remained significantly elevated until two months later while in the DES group it was only significantly elevated at four hours (S1 File). Raw data are presented in supplementary material. The number of patients in the analysis of various biomarkers appears to be fluctuating. This is because our analysis involved paired samples and patients with missing biomarkers even at a single time-point had to be excluded.

## Discussion

There are only limited data about the role of monocytes following PCI. Fakuda et al were the first to link circulating monocytes with in-stent neointimal hyperplasia. They demonstrated that circulating monocytes increase after PCI with bare metal stent, peak at two days and the maximum monocyte level positively correlated with in-stent neointimal volume [8]. Our study is novel as we demonstrated the monocyte subset response after elective PCI in the modern era. We have demonstrated that the population of intermediate monocytes decreased in the immediate post-PCI period (four hours), recovered at two weeks and then significantly increased further at two months. The classical monocytes appeared to follow the opposite pattern to intermediate monocytes while the non-classical monocytes did not change significantly, suggesting that there was a shift from classical to intermediate monocytes and vice versa. Subgroup analysis showed few differences in the monocyte response between DCB and DES groups, most notable being that in the DES group the intermediate monocytes are significantly increased at two months compared to baseline while in the DCB group they were not. The fact that intermediate monocytes, a highly pro-atherosclerotic monocyte subset, remained persistently elevated two months after elective, uncomplicated PCI is a concern and requires further validation and investigation. Our study included a small number of patients and should be regarded as hypothesis generating. However, a possible explanation could be that the stent as a metallic foreign material represents a persistent stimulus for the monocyte response. This hypothesis is consistent with previous studies which have shown that platelet and neutrophil activation is greater after stenting compared to balloon angioplasty only [12].

The current classification of monocytes, which introduced the intermediate subset, was established in 2010 [9]. Since then, there has been an increasing interest about their role in cardiovascular diseases. Over the last ten years, studies have shown that intermediate monocytes are an independent predictor of cardiovascular events in stable patients [10,15]. In a large prospective study of almost 1000 patients being referred for elective coronary angiography, intermediate monocytes were the only subset independently predictive of adverse cardiovascular events [10]. In addition, stable angina patients with elevated levels of the highly proatherogenic lipoprotein (a) (Lp(a)) have significantly elevated levels of intermediate monocytes and oxidized phospholipids (OxPL) [16]. The biomarker OxPL/apoB (oxidized phospholipids on apolipoprotein B-100) correlates with intermediate monocytes but not with the other monocyte subsets [16].

The predictive role of intermediate monocytes has also been demonstrated in patients with acute coronary syndrome. Intermediate and non-classical monocytes are significantly increased in patients with unstable angina when compared with stable patients [17]. Furthermore, in unstable angina patients with intermediate-to-high cardiovascular risk (as determined by GRACE score) intermediate monocytes are increased independently of traditional risk factors [17]. In patients with STEMI, intermediate monocytes have unique functional characteristics, increase significantly in the early stages and are independent predictors of cardiovascular events at two years [18–20]. Beyond the context of coronary artery disease, intermediate monocytes significantly increase in advanced stages of peripheral vascular disease and are associated with risk of restenosis following peripheral vascular angioplasty [21,22]. In addition, they have been shown to be independently associated with and be linked to the pathogenesis of atrial fibrillation [23].

Monocytes play a central role in the crosstalk between T-lymphocytes, endothelial cells and smooth muscle cells mediated by cytokines [24]. In this present study, we have demonstrated changes in the gene expression of CD14+ leucocytes, indicating changes in the functional profile of leucocytes. IL18 showed decreased expression at two weeks, CXCR4 and IL1β decreased at two months, while pentraxin 3 increased at two weeks and two months. The decrease of IL18 and IL1β was mainly driven by the DES group. Interestingly, IL1β, CXCR4 and PTX3 had sustained different levels of expression two months later, indicating that the change in gene expression is not a transient response even after uncomplicated PCI for elective patients. IL18 is a pleiotropic proinflammatory cytokine playing roles in neointimal formation, smooth muscle cell migration as well as plaque vulnerability. Higher levels of IL18 have been associated with increased risk of in-stent restenosis [25]. Monocytes are one of the main producers of IL1β, a cytokine that is known to induce an inflammatory response in vessel wall and is closely related to atherosclerosis as shown by the recent CANTOS trial [26]. Expression of CXCR4 receptor has recently been shown to be atheroprotective by a variety of mechanisms such as maintaining arterial integrity, preserving endothelial function and promoting a normal contractility of smooth muscle cells [27]. PTX3 is produced locally at sites of inflammation by a number of cells such as monocytes, endothelial cells, smooth muscle cells, dendritic cells and fibroblasts [28]. It increases after elective PCI and the post-PCI levels are predictive of major adverse cardiovascular events [29].

The humoral inflammatory response post-PCI has been studied extensively over the last few decades. Our study is the first to demonstrate that TNFα remains significantly elevated two months after elective uncomplicated PCI. TNFα is a key pro-inflammatory cytokine acting locally at sites of vascular injury such as PCI. It promotes the interaction between circulating leucocytes and endothelial cells [30]. Clinical and pre-clinical data have shown that it is associated with restenosis [31]. Our finding that IL6 peaks at four hours post-PCI and remains significantly elevated up to two months later is consistent with a large previous study that had showed that IL6 levels peak 24hours post-PCI and return to baseline by three months [32]. IL6 is a multifunctional cytokine known to induce other acute phase proteins and play a central role in inflammation and tissue injury. It increases immediately post-PCI in the coronary sinus circulation and correlates positively with late loss index at six months [33].

Targeting the residual inflammation after PCI is one of the main avenues current cardiovascular research pursues in order to improve patient outcomes [5,34]. In this study, we have demonstrated that intermediate monocytes, a highly proatherogenic monocyte subset, remain significantly elevated two months following elective, uncomplicated PCI. This might be a potential target of the immune system that could lead to improved patient outcomes. Furthermore, we have demonstrated differences in the elicited inflammatory response between two different, modern PCI strategies. It might be that the PCI strategy could be one of the ways to modulate the elicited inflammatory response post-PCI and improve patient outcomes.

### Limitations

Our study has a number of limitations. First, we recruited a small number of patients and few patients were lost to follow up. This makes it difficult to draw definite conclusions about any subgroup comparisons. Second, we were only able to follow up the patients up to two months post PCI. Longer follow up would provide additional information about the monocyte response and strengthen the value of our findings. Third, we only used CD14 and CD16 markers but not HLA-DR markers to identify monocytes. Therefore, it is possible that not all monocytes were identified. However, it is unlikely that the intermediate monocytes, which is the main finding of our study, were affected by this. Fourth, we studied a limited number of genes and inflammatory mediators. A more comprehensive gene expression analysis would provide a greater understanding of the changes of various monocyte subsets. The expression profile represented a pool of all CD14+ leucocytes rather than specific monocytes subsets. Future studies focusing on specific subsets could help gain insight of the gene alterations that take place at the monocyte subset level. Finally, the decision between DCB or DES was not randomised, but it was left entirely at the discretion of the operator. Previous work from our group has demonstrated that routine DCB-only angioplasty is safe in elective and STEMI patients and discussed the rational of when an operator might choose to use a DCB or a stent. In any case, given the very similar characteristics in this cohort, we do not believe that the differences seen can be explained by anything else other than the percutaneous intervention itself [35–38].

## Conclusion

In conclusion, our study explored the monocyte response following elective PCI. We have demonstrated that the intermediate monocytes significantly decreased acutely at four hours, recovered at two weeks and significantly increased at two months. Subgroup analysis demonstrated that intermediate monocytes were significantly elevated two months post PCI in the DES group but not in the DCB group. Analysis of pooled CD14+ leucocytes has demonstrated that the monocyte response was accompanied by changes in gene expression of important inflammatory mediators, which were maintained up to two months. Finally, we have described the inflammatory biomarker response after elective PCI and demonstrated that some important inflammatory mediators (TNFα, IL6) remained significantly elevated up to two months. Future, larger studies should focus on the differences between DCB and DES in terms of monocyte response, monocyte subset gene expression and also inflammatory biomarkers.

## Supporting information

S1 Fig (TIF)

S1 File Supplementary material for the study. (DOCX)
