# Supplementary figures and images for: Circulating intermediate monocytes CD14++CD16+ are increased after elective percutaneous coronary intervention
Source: PLoS One. 2023 Dec 14;18(12):e0294746. doi: 10.1371/journal.pone.0294746 (PMC10721025; doi:10.1371/journal.pone.0294746)

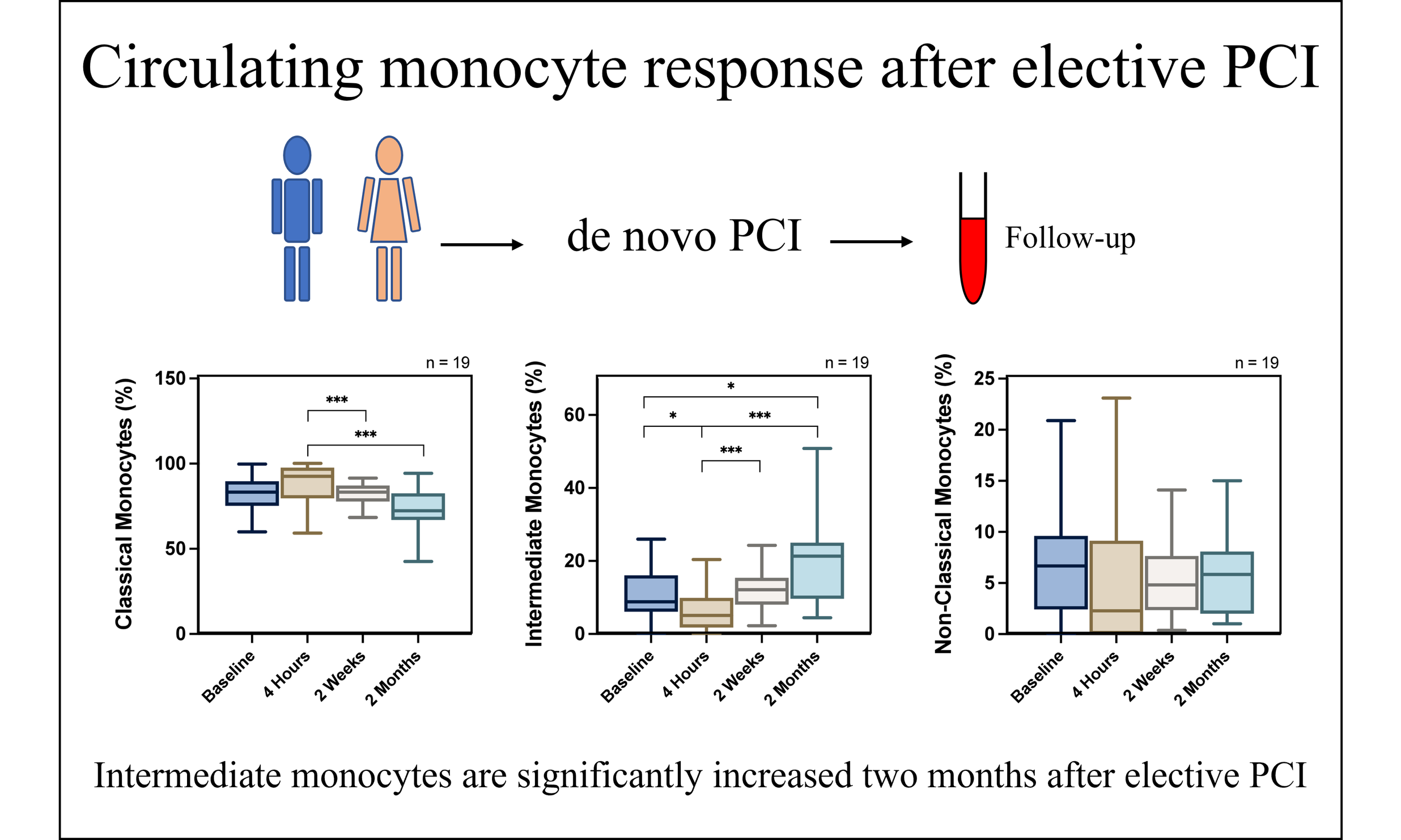

Supplement: S1 Fig — (TIF) [file pone.0294746.s001.tif]
