# Supplementary material for: Circulating intermediate monocytes CD14++CD16+ are increased after elective percutaneous coronary intervention
Source: PLoS One. 2023 Dec 14;18(12):e0294746. doi: 10.1371/journal.pone.0294746 (PMC10721025; doi:10.1371/journal.pone.0294746)
Supplement: S1 File — (DOCX) [file pone.0294746.s002.docx]

**Circulating intermediate monocytes CD14++CD16+ are increased after elective percutaneous coronary intervention**

Supplement to article by Merinopoulos et al.

Flow cytometry

Fixed cells were sieved using 70μm cell strainer and centrifuged at 1500RPM for 5min.The supernatant was discarded and the cells re-suspended in 500μl MACS buffer. 250μl were transferred to an Eppendorf tube and 1μl of CD14 and CD16 antibody were added. Further 250μl PBS were added to the solution before flow cytometry analysis. Fluorescence Minus One (FMO) controls were used to identify positive/negative gating boundaries.

FlowJo version 10 was used for analysis of flow cytometry results. All the FCS files from the flow cytometer were uploaded to flowjo and grouped together per patient. We followed the steps below to identify the monocyte populations of interest.

1. Cells visualized on a forward scatter area FSC(A) / forward scatter height FSC(H) plot, in order to select cells that have an equal area and height and exclude debris and clumps.

2. Selected cells were visualized on a FSC(A)/ side scatter SSC(A) plot. Monocyte populations were selected based on their forward and side scatter properties and the majority of lymphocytes, natural killer cells and granulocytes were excluded.

3. Selected cells were visualized on a CD16 / CD14 plot and monocytes were selected based on their characteristic ‘┐‘ shape.

4. Selected monocytes were re-displayed on a CD16 / CD14 plot to gate the monocyte subpopulations.

Example of monocyte gating strategy. A) FSC(A) vs FSC(H) plot gating cells according to area and height and removing clumps and debris. B) FSC(A) vs SSC(A) plot broadly selecting monocytes based on their SSC/FSC properties C) FITC (A) vs APC(A) plot selecting monocytes according to their characteristic *‘┐‘ shape D) CD14 vs CD16 plot*

A) B)


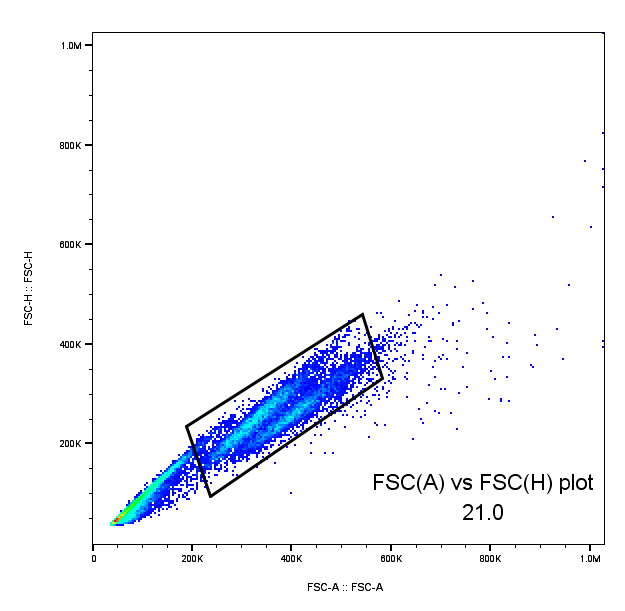

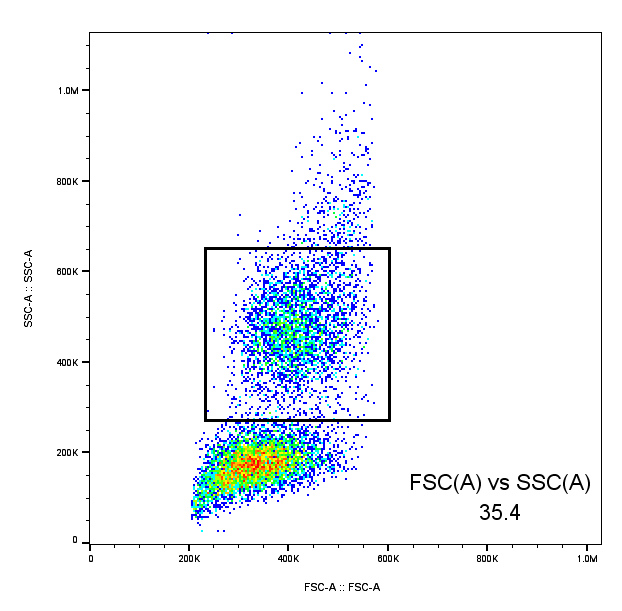


C) D)


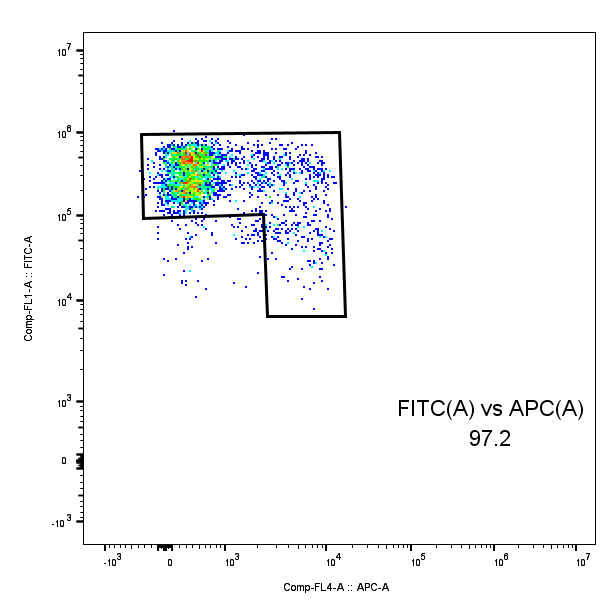

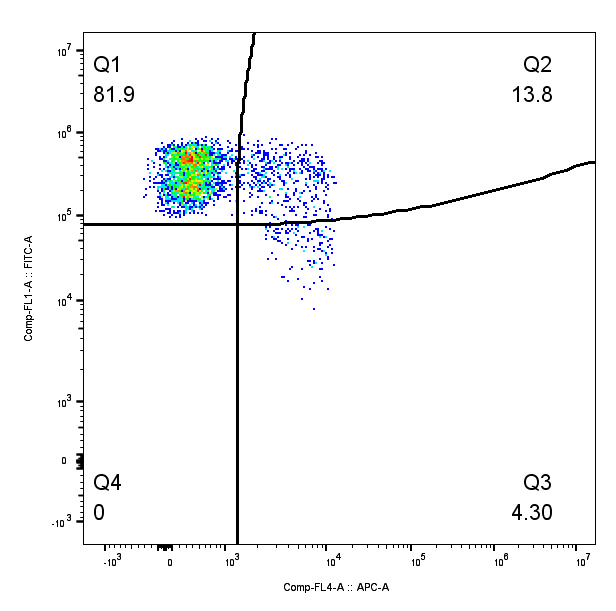


Supplementary figure 1 demonstrates the monocyte response for the classical, intermediate and non-classical monocyte subsets after elective angioplasty with drug coated balloon (A) or drug eluting stent (B). * p<0.05 ** p<0.01 *** p<0.001

A)


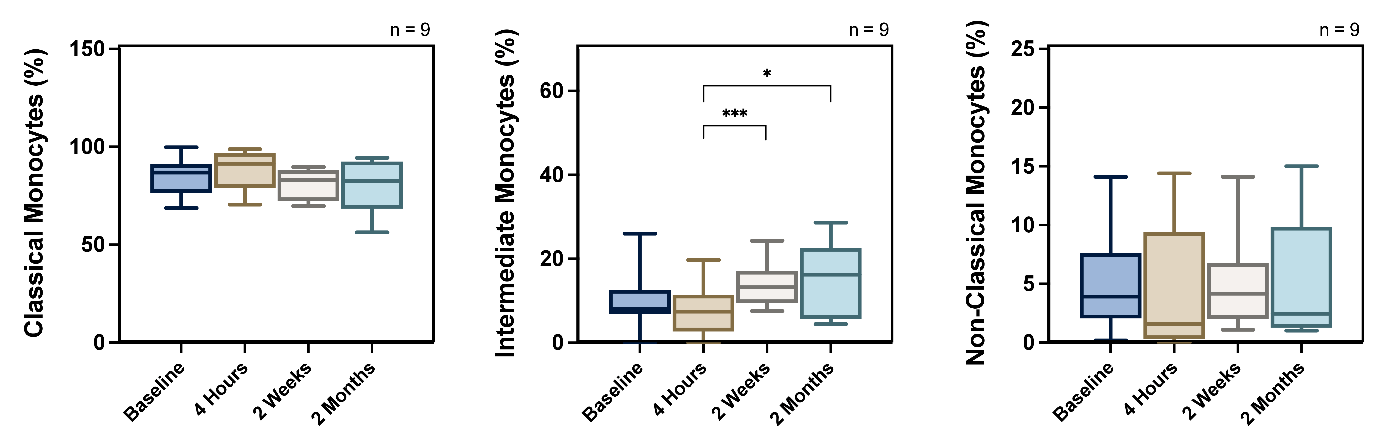


B)


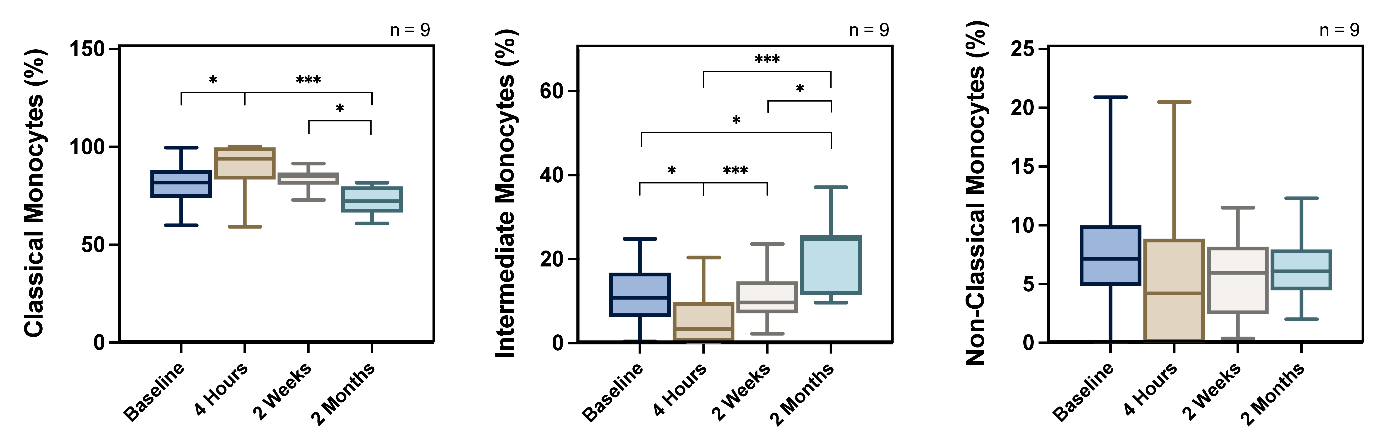


Supplementary figure 2 shows the gene expression (fold change compared to baseline) of CD14+ leucocytes following elective angioplasty with drug coated balloon (A) or drug eluting stent (B). * p<0.05 ** p<0.01 *** p<0.001

A)


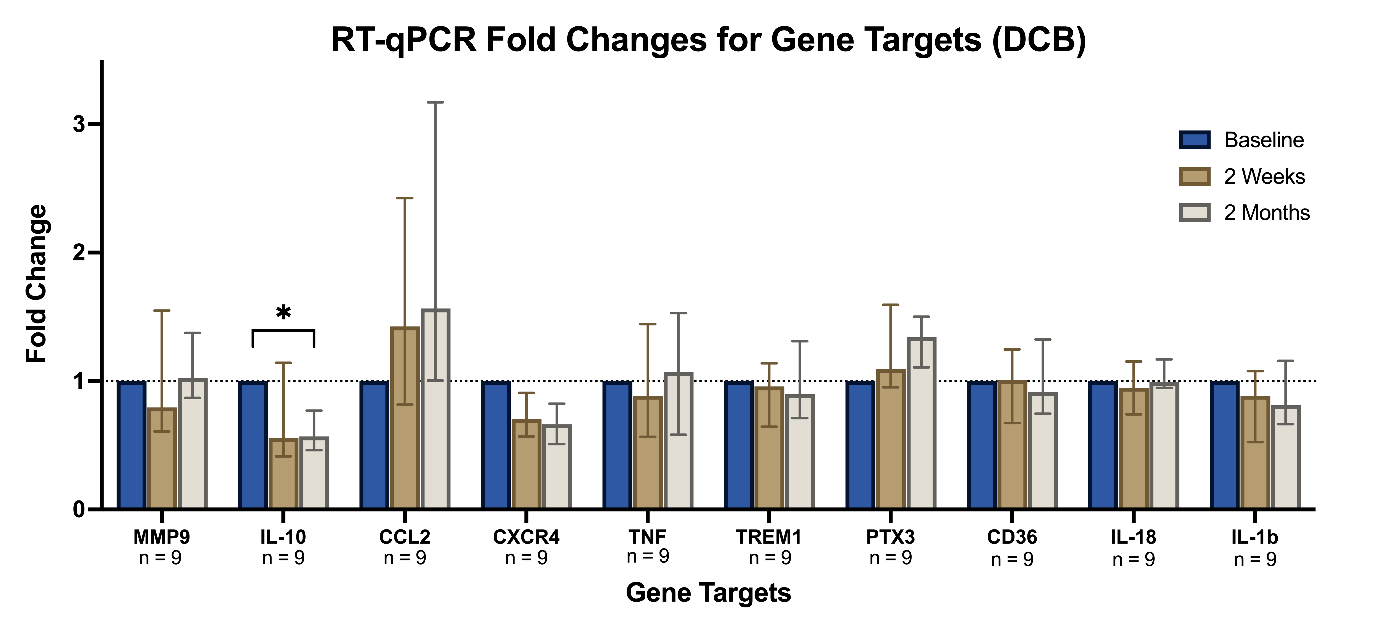


B)


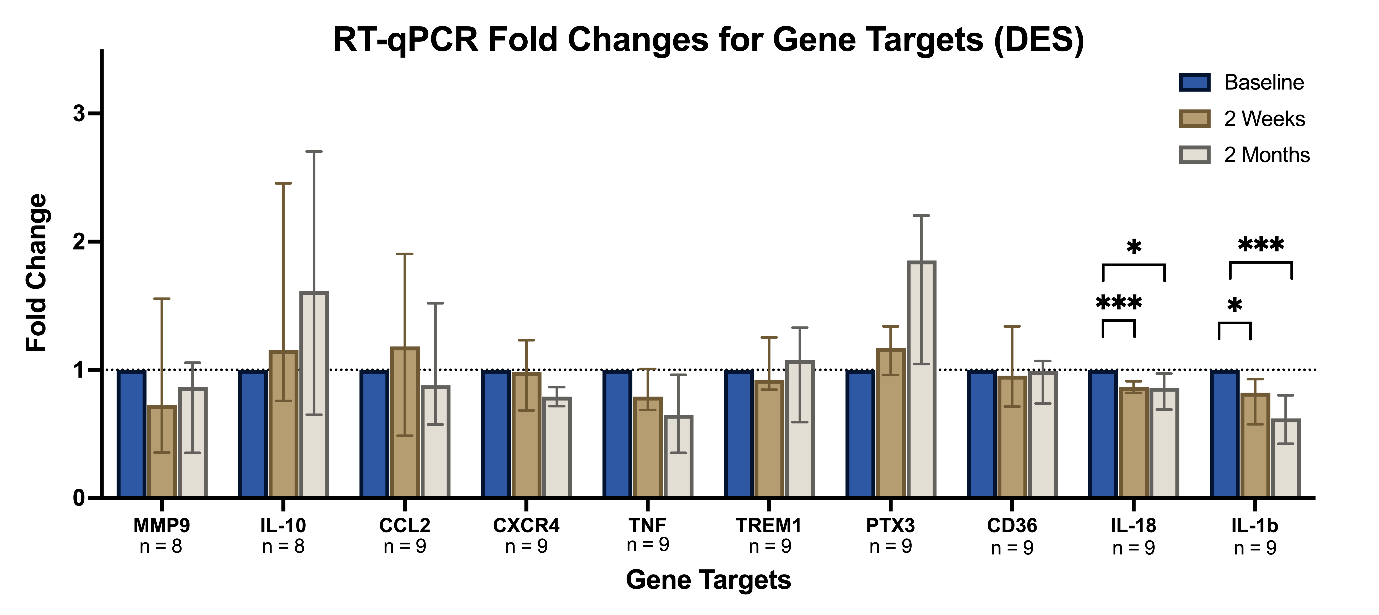


Supplementary figure 3 shows the inflammatory response after elective angioplasty with drug coated balloon (A) or drug eluting stent (B). * p<0.05 ** p<0.01 *** p<0.001

A)


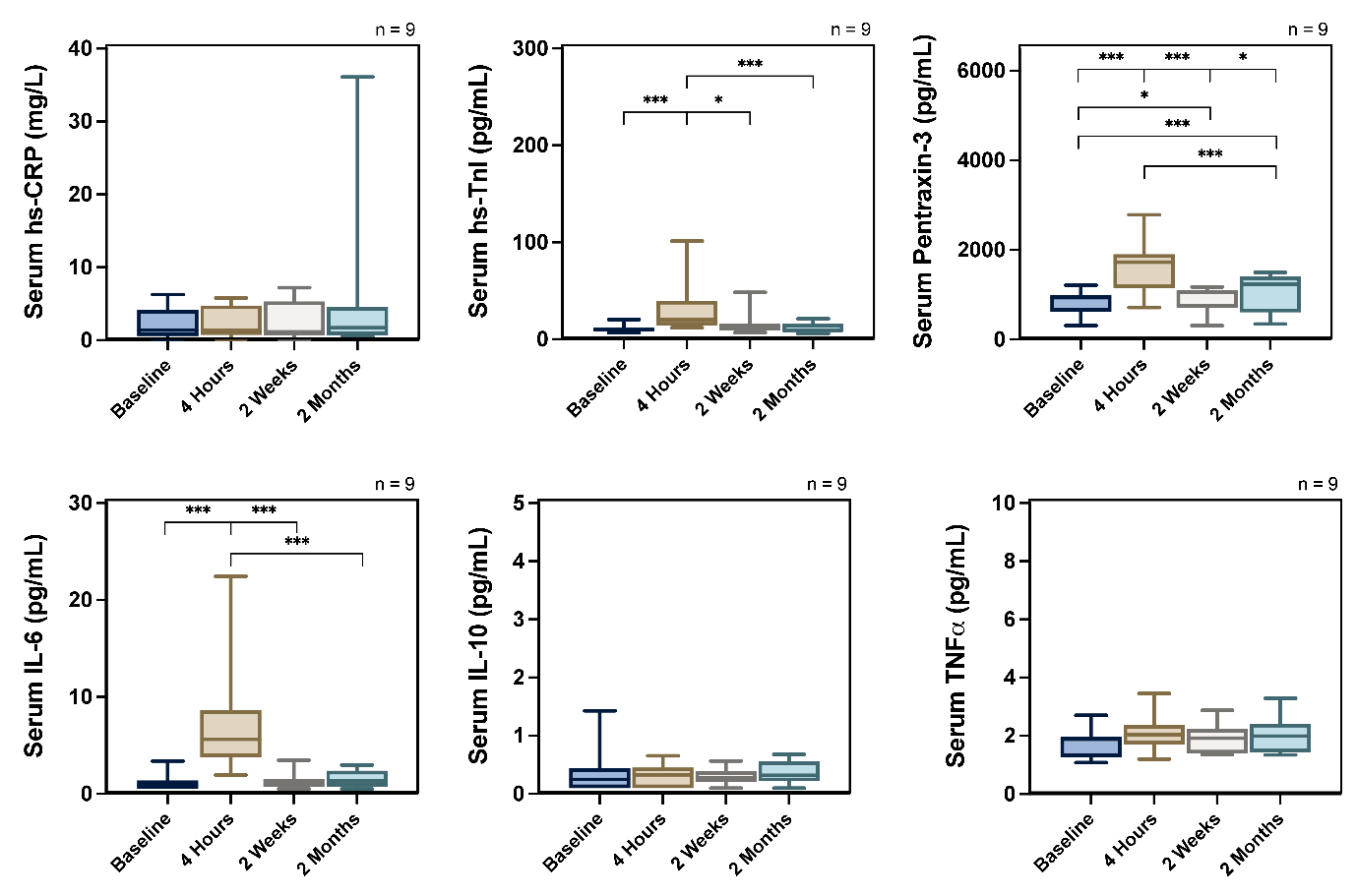


B)


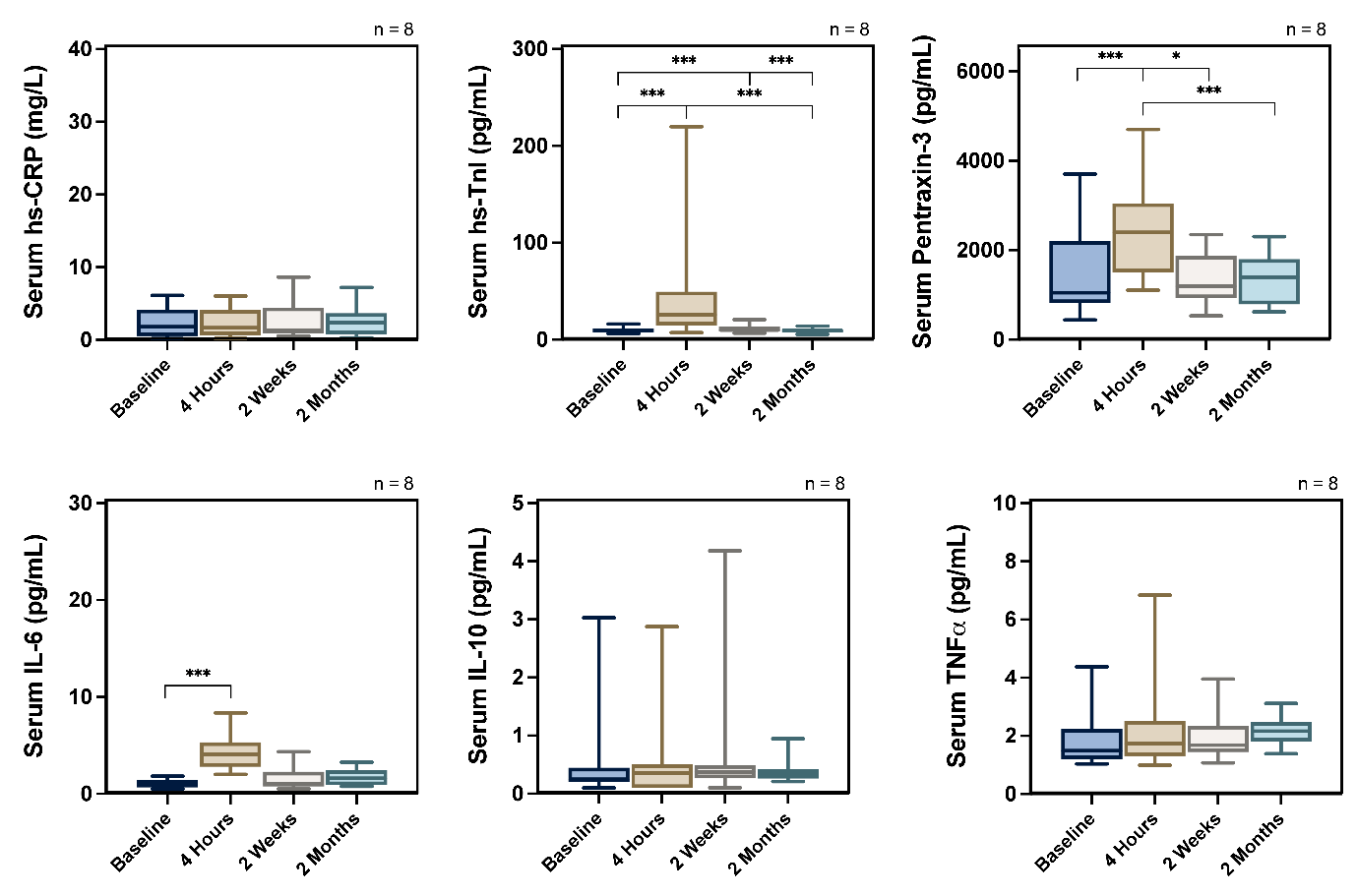


Supplementary table 1: Primer sequences used for RT-qPCR

| **Primer** | **Forward** | **Reverse** |
| --- | --- | --- |
| GADPH (Housekeeper) | 5’ – ACAGTTGCCATGTAGACC | 5’ - TTGAGCAGGGTACTTTA |
| MMP9 | 5’ - AAGGATGGGAAGTACTGG | 5’ - GCCCAGAGAAGAAGAAAAG |
| IL-10 | 5’ - GCCTTTAATAAGCTCCAAG | 5’ - ATCTTCATTGTCATGTAGGC |
| CCL2 | 5’ - AGACTAACCCAGAAACATCC | 5’ - ATTGATTGCATCTGGCTG |
| CXCR4 | 5’ - GGTGGTCTATGTTGGCGTCT | 5’ - CTCACTGACGTTGGCAAAGA |
| TNFα | 5’ - CTCAGCCTCTTCTCCTTC | 5’ - AGAAGATGATCTGACTGCC |
| TREM1 | 5’ - ACAGATATCATCAGGGTTCC | 5’ - CCTAGGGTACAAATGACCTC |
| PTX3 | 5’ - AGAGAGAGTTGAGACCAATC | 5’ - AAACAATTGTCCCTCTGTTC |
| CD36 | 5’ - AGCTTTCCAATGATTAGACG | 5’ – CAACTGGCATTAGAATACCTC |
| IL18 | 5’ – CAGCCGCTTTAGCAGCCA | 5’ - CAAGGAATTGTCTCCCAGTGC |

Supplementary table 2: Baseline medications

| **Medications** | **All patients (N=26)** | **DCB group (N=10)** | **DES group (N=15)** | **P value** |
| --- | --- | --- | --- | --- |
| Aspirin | 25 (96.2%) | 9 (90%) | 15 (100%) | 0.21 |
| Clopidogrel / Ticagrelor | 26 (100%) | 10 (100%) | 15 (100%) | n/a |
| Statin | 24 (92.3%) | 9 (90%) | 14 (93.3%) | 0.76 |
| Betablocker | 18 (69.2%) | 7 (70%) | 10 (66.7%) | 0.86 |
| Nitrate | 8 (30.8%) | 4 (40%) | 4 (26.7%) | 0.48 |
| ACE/ARB | 17 (65%) | 6 (60%) | 11 (73.3%) | 0.48 |
| Calcium channel blocker | 8 (30/8%) | 4 (40%) | 4 (26.7%) | 0.48 |
| Ivabradine | 1 (3.8%) | 1 (10%) | 0 | 0.21 |
| Ranolazine | 6 (23.1%) | 4 (40%) | 2 (13.3%) | 0.13 |

Supplementary table 3: Procedural characteristics

|  | **All patients (N=26)** | **DCB group**  **N=10)** | **DES group**  **(N=15)** | **P value** |
| --- | --- | --- | --- | --- |
| Radial access | 24 (96%) | 10 (100%) | 14 (93.3%) | 0.41 |
| Number of devices (stents or drug coated balloons) used | | | | |
| 1 device  2 devices  3 devices | 19 (76%)  5 (20%)  1 (4%) | 6 (60%)  3 (30%)  1 (10%) | 13 (86.7%)  2 (13.3%)  0 (0%) | 0.24 |
| Mean device diameter (mm) | 3.2 (0.55) | 3.1 (0.46) | 3.3 (0.59) | 0.31 |
| Mean device length (mm) | 29.3 (14.3) | 34 (20.1) | 26.2 (8.2) | 0.27 |

Supplementary table 4: Biomarkers, monocyte subsets and gene expression at baseline, 4hours, 2 weeks and 2 months post PCI

| **Characteristic** | **Overall**, N = 26^1^ | **DCB**, N = 10 | **DES**, N = 15 | **p-value**^2^ |
| --- | --- | --- | --- | --- |
| E_hsCRP_Baseline, Median (IQR) | 1.43 (0.57 – 3.83) | 1.39 (0.67 – 3.48) | 1.82 (0.60 – 4.02) | 0.85 |
| E_hsCRP_4 Hours, Median (IQR) | 1.43 (0.71 – 4.12) | 1.35 (0.79 – 4.00) | 1.68 (0.67 – 3.98) | 0.95 |
| E_hsCRP_2 Weeks, Median (IQR) | 1.32 (0.88 – 4.47) | 1.16 (0.84 – 4.89) | 1.34 (1.06 – 3.78) | 0.51 |
| E_hsCRP_2 Months, Median (IQR) | 1.70 (0.81 – 3.02) | 1.7 (0.8 – 2.4) | 2.4 (0.9 – 3.6) | 0.93 |
| E_hsTropI_Baseline, Median (IQR) | 9.35 (7.80 – 11.20) | 9.90 (8.38 – 11.35) | 9.30 (7.80 – 10.75) | 0.58 |
| E_hsTropI_4 Hours, Median (IQR) | 21 (14 – 38) | 20 (14 – 28) | 26 (16 – 39) | 0.63 |
| E_hsTropI_2 Weeks, Median (IQR) | 10.0 (8.8 – 15.4) | 11.0 (9.2 – 15.7) | 9.9 (8.7 – 12.2) | 0.49 |
| E_hsTropI_2 Months, Median (IQR) | 9.2 (7.3 – 10.9) | 9.2 (8.4 – 13.9) | 9.7 (7.4 – 9.9) | 0.60 |
| E_Pent3_Baseline, Median (IQR) | 975 (735 – 1,303) | 936 (682 – 982) | 1,051 (877 – 1,982) | 0.071 |
| E_Pent3_4 Hours, Median (IQR) | 1,925 (1,329 – 2,620) | 1,726 (1,249 – 1,886) | 2,398 (1,686 – 2,969) | **0.031** |
| E_Pent3_2 Weeks, Median (IQR) | 1,090 (845 – 1,559) | 1,051 (779 – 1,092) | 1,194 (981 – 1,872) | 0.055 |
| E_Pent3_2 Months, Median (IQR) | 1,234 (753 – 1,460) | 1,230 (737 – 1,380) | 1,399 (852 – 1,788) | 0.22 |
| E_IL6_Baseline, Median (IQR) | 0.93 (0.55 – 1.40) | 0.88 (0.50 – 1.35) | 0.94 (0.74 – 1.43) | 0.59 |
| E_IL6_4 Hours, Median (IQR) | 4.47 (3.10 – 6.39) | 5.63 (4.03 – 7.86) | 4.09 (2.88 – 4.90) | 0.14 |
| E_IL6_2 Weeks, Median (IQR) | 1.03 (0.80 – 1.75) | 1.02 (0.84 – 1.24) | 1.03 (0.80 – 2.01) | 0.76 |
| E_IL6_2 Months, Median (IQR) | 1.50 (0.91 – 2.01) | 1.36 (0.91 – 2.06) | 1.61 (0.98 – 1.96) | 0.60 |
| E_IL10_Baseline, Median (IQR) | 0.25 (0.13 – 0.39) | 0.25 (0.13 – 0.37) | 0.25 (0.21 – 0.42) | 0.62 |
| E_IL10_4 Hours, Median (IQR) | 0.33 (0.10 – 0.44) | 0.33 (0.14 – 0.42) | 0.35 (0.13 – 0.48) | 0.79 |
| E_IL10_2 Weeks, Median (IQR) | 0.34 (0.24 – 0.40) | 0.28 (0.21 – 0.38) | 0.37 (0.29 – 0.46) | 0.26 |
| E_IL10_2 Months, Median (IQR) | 0.32 (0.26 – 0.42) | 0.32 (0.22 – 0.55) | 0.34 (0.26 – 0.41) | 0.73 |
| E_TNFa_Baseline, Median (IQR) | 1.73 (1.27 – 2.00) | 1.88 (1.41 – 1.97) | 1.49 (1.24 – 2.12) | 0.81 |
| E_TNFa_4 Hours, Median (IQR) | 1.86 (1.34 – 2.32) | 2.04 (1.79 – 2.32) | 1.74 (1.34 – 2.33) | 0.47 |
| E_TNFa_2 Weeks, Median (IQR) | 1.91 (1.45 – 2.22) | 1.92 (1.52 – 2.16) | 1.68 (1.49 – 2.28) | 0.85 |
| E_TNFa_2 Months, Median (IQR) | 2.05 (1.55 – 2.35) | 1.99 (1.48 – 2.39) | 2.16 (1.98 – 2.31) | 0.73 |
| Classical_Mo_Baseline, Median (IQR) | 83 (76 – 89) | 87 (79 – 90) | 82 (74 – 88) | 0.24 |
| Classical_Mo_4 Hours, Median (IQR) | 92 (81 – 97) | 91 (81 – 96) | 94 (84 – 99) | 0.41 |
| Classical_Mo_2 Weeks, Median (IQR) | 83.2 (79.4 – 86.7) | 83.0 (74.8 – 86.8) | 85.0 (81.4 – 86.6) | 0.43 |
| Classical_Mo_2 Months, Median (IQR) | 72 (67 – 82) | 82 (69 – 91) | 72 (67 – 78) | 0.14 |
| Intermediate_Mo_Baseline, Median (IQR) | 9 (7 – 15) | 8 (7 – 11) | 11 (7 – 16) | 0.50 |
| Intermediate_Mo_4 Hours, Median (IQR) | 5.1 (2.0 – 9.5) | 7.4 (3.2 – 9.9) | 3.3 (0.9 – 8.8) | 0.31 |
| Intermediate_Mo_2 Weeks, Median (IQR) | 12.1 (8.4 – 15.1) | 13.2 (10.1 – 15.8) | 9.7 (7.5 – 13.6) | 0.16 |
| Intermediate_Mo_2 Months, Median (IQR) | 21 (10 – 25) | 16 (7 – 21) | 25 (13 – 25) | 0.093 |
| Non_Classical_Mo_Baseline, Median (IQR) | 6.7 (2.7 – 9.3) | 3.9 (2.3 – 6.9) | 7.2 (5.2 – 9.7) | 0.16 |
| Non_Classical_Mo_4 Hours, Median (IQR) | 2.3 (0.0 – 8.7) | 1.6 (0.4 – 6.5) | 4.2 (0.0 – 8.6) | 0.98 |
| Non_Classical_Mo_2 Weeks, Median (IQR) | 4.8 (2.5 – 7.5) | 4.2 (2.3 – 6.3) | 6.0 (2.7 – 7.8) | 0.50 |
| Non_Classical_Mo_2 Months, Median (IQR) | 5.8 (2.2 – 7.9) | 2.5 (1.3 – 8.6) | 6.1 (5.8 – 7.8) | 0.30 |
| PCR_MMP9_Baseline | 1 | 1 | 1 |  |
| PCR_MMP9_2 Weeks, Median (IQR) | 0.77 (0.42 – 1.62) | 0.79 (0.61 – 1.55) | 0.72 (0.36 – 1.56) | 0.51 |
| PCR_MMP9_2 Months, Median (IQR) | 0.95 (0.50 – 1.36) | 1.02 (0.87 – 1.38) | 0.87 (0.35 – 1.06) | 0.28 |
| PCR_IL10_Baseline | 1 | 1 | 1 |  |
| PCR_IL10_2 Weeks, Median (IQR) | 0.96 (0.57 – 1.66) | 0.56 (0.41 – 1.14) | 1.16 (0.76 – 2.46) | **0.031** |
| PCR_IL10_2 Months, Median (IQR) | 0.75 (0.54 – 0.91) | 0.57 (0.46 – 0.77) | 1.62 (0.65 – 2.70) | **0.046** |
| PCR_CCL2_Baseline | 1 | 1 | 1 |  |
| PCR_CCL2_2 Weeks, Median (IQR) | 1.10 (0.67 – 1.94) | 1.43 (0.82 – 2.43) | 1.18 (0.49 – 1.90) | 0.63 |
| PCR_CCL2_2 Months, Median (IQR) | 1.00 (0.63 – 2.37) | 1.57 (1.00 – 3.17) | 0.88 (0.57 – 1.52) | 0.26 |
| PCR_CXCR4_Baseline | 1 | 1 | 1 |  |
| PCR_CXCR4_2 Weeks, Median (IQR) | 0.83 (0.64 – 1.12) | 0.71 (0.57 – 0.91) | 0.98 (0.68 – 1.23) | 0.17 |
| PCR_CXCR4_2 Months, Median (IQR) | 0.72 (0.52 – 0.86) | 0.66 (0.51 – 0.82) | 0.79 (0.72 – 0.87) | 0.44 |
| PCR_TNF_Baseline | 1 | 1 | 1 |  |
| PCR_TNF_2 Weeks, Median (IQR) | 0.78 (0.52 – 1.13) | 0.89 (0.57 – 1.44) | 0.79 (0.69 – 1.01) | 0.80 |
| PCR_TNF_2 Months, Median (IQR) | 0.66 (0.36 – 1.10) | 1.07 (0.58 – 1.53) | 0.65 (0.35 – 0.96) | 0.26 |
| PCR_TREM1_Baseline | 1 | 1 | 1 |  |
| PCR_TREM1_2 Weeks, Median (IQR) | 0.89 (0.67 – 1.21) | 0.96 (0.65 – 1.14) | 0.92 (0.85 – 1.25) | 0.40 |
| PCR_TREM1_2 Months, Median (IQR) | 0.90 (0.62 – 1.32) | 0.90 (0.71 – 1.31) | 1.08 (0.59 – 1.33) | 0.73 |
| PCR_PTX3_Baseline | 1 | 1 | 1 |  |
| PCR_PTX3_2 Weeks, Median (IQR) | 1.13 (0.94 – 1.36) | 1.10 (0.95 – 1.59) | 1.17 (0.96 – 1.34) | 0.80 |
| PCR_PTX3_2 Months, Median (IQR) | 1.38 (1.08 – 1.98) | 1.34 (1.11 – 1.50) | 1.85 (1.05 – 2.20) | 0.73 |
| PCR_CD36_Baseline | 1 | 1 | 1 |  |
| PCR_CD36_2 Weeks, Median (IQR) | 0.90 (0.65 – 1.27) | 1.01 (0.67 – 1.25) | 0.96 (0.71 – 1.34) | >0.99 |
| PCR_CD36_2 Months, Median (IQR) | 0.91 (0.71 – 1.12) | 0.91 (0.75 – 1.32) | 0.99 (0.74 – 1.07) | 0.80 |
| PCR_IL18_Baseline | 1 | 1 | 1 |  |
| PCR_IL18_2 Weeks, Median (IQR) | 0.88 (0.74 – 0.97) | 0.95 (0.74 – 1.15) | 0.87 (0.82 – 0.91) | 0.47 |
| PCR_IL18_2 Months, Median (IQR) | 0.95 (0.86 – 1.04) | 0.99 (0.95 – 1.17) | 0.86 (0.69 – 0.97) | 0.14 |
| PCR_IL1B_Baseline | 1 | 1 | 1 |  |
| PCR_IL1B_2 Weeks, Median (IQR) | 0.83 (0.49 – 0.98) | 0.89 (0.52 – 1.08) | 0.82 (0.58 – 0.93) | 0.63 |
| PCR_IL1B_2 Months, Median (IQR) | 0.69 (0.51 – 0.88) | 0.81 (0.67 – 1.16) | 0.62 (0.42 – 0.80) | 0.30 |
